# Critical methodological concerns regarding the validation of NanOx model in helium SOBP irradiation

**DOI:** 10.1016/j.phro.2026.101090

**Published:** 2026-10-01

**Authors:** Ehsan Khodamoradi

**Affiliations:** Kermanshah University of Medical Sciences, Kermanshah, Iran

While the study by Berger et al. addresses a critical gap in helium ion radiobiology, two fundamental methodological flaws severely undermine the validity of their conclusions.

**First, Circular Validation:** The authors explicitly state that *"the parameters of the NanOx model were tuned based on experimental values of the alpha coefficient for protons and carbon ions of different energies"* [1]. The optimization of the ELLF parameters (h = 84,246.7 Gy, z₀ = 16,279.7 Gy, and sigma = 165.016 Gy) was performed using experimental data from reference [2] (Friedrich et al.) for the **exact same SQ20B cell line, as detailed in the ELLF determination study** [3]. The authors then used this identical cell line to “validate” the model's predictions against helium ion irradiation. This does not constitute independent validation; it is merely an interpolation exercise within a parameter space constrained by the same biological system. A scientifically rigorous validation requires an independent dataset–whether from a different cell line or experimental condition–that played no role in the initial parameter tuning. Without this independence, the reported agreement largely reflects the model's fitting capacity rather than its true predictive power. As the authors themselves acknowledge, the model for helium ions *“remains largely constrained by indirect calibration data and limited helium-specific radiobiological measurements”*–this statement itself testifies to the model's heavy reliance on indirect calibration data.

**Second, Acknowledged Systematic Dosimetric Error (LET Quenching):** In Section 2.1, the authors explicitly state that for the HDV2 radiochromic films, *“the potential LET dependence of the film response, which may affect absolute dose accuracy, was not corrected for and is considered a limitation of this validation approach”*. This omission is critical, particularly in the distal SOBP region where LET peaks. Film quenching at high LET is a well-documented phenomenon in radiochromic film dosimetry for heavy-ion beams [4]. Multiple studies have demonstrated that the response of Gafchromic films depends on both dose and LET, with film quenching varying nonlinearly as a function of LET. This acknowledged systematic error can lead to an underestimation of absorbed dose by 10–20% in high-LET regions. Given that survival curves and RBE calculations are exquisitely sensitive to absolute dose accuracy, this directly biases the quantitative comparison between experimental data and NanOx predictions, especially at the distal edge where the largest model-experiment discrepancy (37%) is observed. Recent work on LET-independent calibration of radiochromic films for carbon-ion beams has demonstrated that correcting this effect is essential for accurate dosimetry in heavy-ion radiotherapy.

Consequently, due to these two irreducible flaws–**the lack of data-independent validation** and **an acknowledged systematic error in dose quantification**–the authors' conclusion that *“these results support further validation of the model in a broader range of cell lines and helium ion irradiation configurations”* is not merely premature but misleading. The logical implication of their own findings points toward an urgent need for **independent re-parameterization** of the NanOx model specifically for helium ions, using quenching-corrected dosimetry, rather than perpetuating a circular validation exercise.

## CRediT authorship contribution statement

**Ehsan Khodamoradi:** Writing – original draft.

## Declaration of competing interest

The authors declare that they have no known competing financial interests or personal relationships that could have appeared to influence the work reported in this paper.
